# Rapid Analysis of Biotherapeutics Using Protein A Chromatography Coupled to Orbitrap Mass Spectrometry

**DOI:** 10.1021/acs.analchem.1c02365

**Published:** 2021-09-29

**Authors:** Craig Jakes, Florian Füssl, Izabela Zaborowska, Jonathan Bones

**Affiliations:** † Characterisation and Comparability Laboratory, The National Institute for Bioprocessing Research and Training, Fosters Avenue, Mount Merrion, County Dublin A94 X099, Ireland; ‡ School of Chemical and Bioprocess Engineering, University College Dublin, Belfield, Dublin 4, D04 V1W8, Ireland

## Abstract

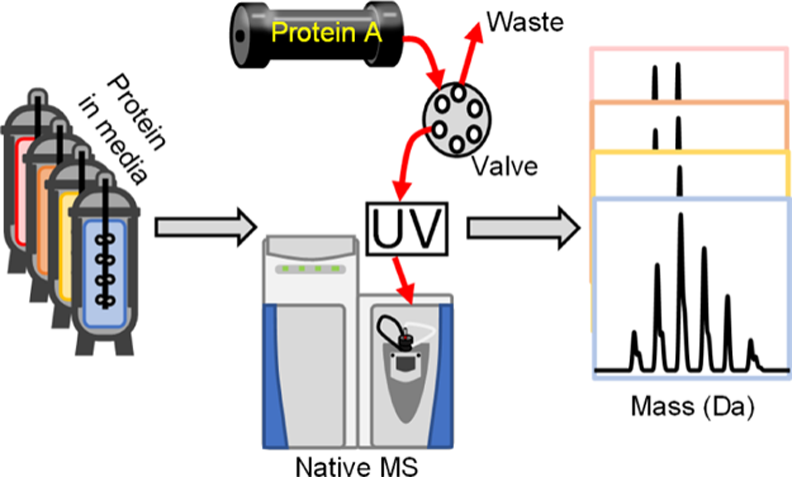

Monoclonal antibodies (mAbs) and related products undergo a wide range of modifications, many of which can often be directly associated to culture conditions during upstream processing. Ideally, such conditions should be monitored and fine-tuned based on real-time or close to real-time information obtained by the assessment of the product quality attribute (PQA) profile of the biopharmaceutical produced, which is the fundamental idea of process analytical technology. Therefore, methods that are simple, quick and robust, but sufficiently powerful, to allow for the generation of a comprehensive picture of the PQA profile of the protein of interest are required. A major obstacle for the analysis of proteins directly from cultures is the presence of impurities such as cell debris, host cell DNA, proteins and small-molecule compounds, which usually requires a series of capture and polishing steps using affinity and ion-exchange chromatography before characterization can be attempted. In the current study, we demonstrate direct coupling of protein A affinity chromatography with native mass spectrometry (ProA-MS) for development of a robust method that can be used to generate information on the PQA profile of mAbs and related products in as little as 5 min. The developed method was applied to several samples ranging in complexity and stability, such as simple and more complex monoclonal antibodies, as well as cysteine-conjugated antibody–drug conjugate mimics. Moreover, the method demonstrated suitability for the analysis of protein amounts of <1 μg, which suggests applicability during early-stage development activities.

The biopharmaceutical industry continues to be dominated by monoclonal antibodies (mAbs), with these molecules expected to hold an estimated share of 20% of the global pharmaceutical market by 2022.^1^ Biopharmaceuticals such as mAbs are produced through genetic engineering of animal cells, such as Chinese hamster ovary (CHO) cells.^2,3^ Typically, the target protein must be purified and undergo full characterization before being released for medicinal use. The most commonly applied method for the purification of mAbs is affinity capture chromatography using protein A from *Staphylococcus aureus*, which has high affinity for immunoglobulin G (IgG) antibodies of subclasses 1, 2 and 4, while only weak interactions are observed with subclass 3.^4^ In protein A chromatography, the fragment crystallizable (Fc) region of the mAb binds to protein A at neutral pH. This selective capture of the protein allows cell culture components such as host cell proteins, DNA, small-molecule components, or other potential contaminants to be removed. The binding of protein A with the mAb has been previously examined through surface tension measurement, mass balance analysis, spectrophotometry and sequencing studies.^5−7^ Sequencing studies found that protein A has five IgG binding domains (E, D, A, B and C), each of which is capable of binding to the Fc region of an IgG. However, a binding study relying on radioiodinated protein A, using both human and rabbit IgG, resulted in a molar binding ratio of 1:1, while UV absorbance and water surface tension analysis have indicated a molar ratio of 1:2 (protein A–IgG). Disagreement exists concerning the stoichiometry of protein A–IgG binding, which was here further investigated using size exclusion chromatography (SEC) coupled to native mass spectrometry (MS) and the pH dependence of complex formation was analyzed by performing the associated SEC–MS experiments under different mobile phase pH conditions.

mAbs can be characterized on different molecular levels such as peptide, subunit, or intact levels using MS. Each level of analysis provides distinct information on the relative abundance and location of post-translational modifications (PTMs), such as glycosylation.^8,9^ Recent technological advances in MS, such as enhanced ion trapping, improved molecular desolvation and declustering and wider applicable mass ranges, have greatly increased the capability of performing intact native protein analysis.^10,11^ While the application of native MS for structural characterization of monoclonal antibodies is not entirely novel, only recently have a number of studies been published, successfully applying traditional liquid chromatography (LC) methodologies directly coupled to MS. For example, SEC has been adapted for coupling with native MS using volatile salts in aqueous mobile phases at neutral pH to promote protein stability. This has allowed for the analysis of several proteins such as myoglobin, cytochrome *C* and mAbs in their native states.^12^ Native SEC–MS has been further employed in the analysis of antibody–drug conjugates (ADCs) and was found to be an effective technique for the quantitation of drug-to-antibody ratios (DARs) with comparable performance to traditional methods such as hydrophobic interaction chromatography (HIC).^13^ Charge variant analysis (CVA) has been successfully adapted for MS analysis through the development of MS-friendly mobile phases that rely on pH- and/or salt-gradient elution of mAb charge variants from cation exchangers.^14,15^ Application of CVA–MS has allowed for the identification of over 100 isoforms in cetuximab, the identification of deamidation and succinimide isoforms in trastuzumab and was successfully employed in the separation and analysis of bispecific antibodies.^16−18^ Native MS directly interfaced to HIC has been achieved through reduction of the salt concentration entering the MS through a flow splitter and has been used for the characterization of mAb mixtures and ADC mimics.^19−21^ Finally, a form of reversed-phase LC has been successfully coupled to native MS for the identification of different DAR species in ADCs.^22^ Albeit having proven to be highly useful for intact protein analysis, common drawbacks with these methods are that they typically require samples that are largely free of cell culture contaminants and analysis times are often in the range of several tens of minutes, which can limit high-throughput screening of large sample sets. Such issues were avoided by a previous attempt to directly couple protein A chromatography to MS which yielded the successful characterization of mAbs and bispecific antibodies.^23^ The presented setup included a flow splitter and the introduction of a makeup flow containing organic solvent which allowed fast run times and high sensitivity but potentially at the cost of reduced method robustness, inability to maintain noncovalent interactions and protein higher order structure when needed.

Here, we present the development of a native protein A chromatography–MS method, which is rapid, robust and can be applied for the analysis of 'fragile' proteins containing a mAb scaffold and can easily be adapted for online process analytical technology (PAT). The method was validated and tested on multiple commercially available mAbs including complex molecules such as cetuximab and for analytes that maintain a higher order structure *via* noncovalent interactions of multiple protein subunits. The method was further employed for the analysis of IgG1 samples derived from cells cultured for up to 10 days in bioreactors under culture conditions varying in the level of dissolved oxygen (DO) and culture temperature to test method applicability in the lab-scale manufacturing setting.

## Experimental Section

### Chemicals and Materials

Ultrapure Optima LC–MS-grade water, LC–MS-grade acetic acid, LC–MS-grade formic acid and phosphate-buffered saline (PBS) were obtained from Fisher Scientific (Dublin, Ireland). Ammonium acetate (99.999% trace metal grade), ammonium formate (≥99.995% trace metal basis) and 4.0 mM l-glutamine were purchased from Sigma-Aldrich (Wicklow, Ireland). Amicon Ultra-0.5 mL centrifugal filters with a 10 kDa molecular weight cutoff size and 0.45 and 0.20 μm poly(vinylidene difluoride) (PVDF) membrane filters were purchased from Merck (Tullagreen, Ireland). BalanCD CHO Growth A was purchased from FUJIFILM Irvine Scientific (Wicklow Ireland). Native *S. aureus* protein A was purchased from Bio-Rad, (Accuscience, Ireland).

### Samples and Sample Preparation

IgG1 monoclonal antibodies (bevacizumab, rituximab, infliximab, trastuzumab, and cetuximab) used in this study were kindly provided by the hospital pharmacy unit of the University Hospital of San Cecilio in Granada, Spain. The ADC mimic (MSQC8) was purchased from Sigma-Aldrich (Wicklow, Ireland) and was prepared according to the manufacturer’s protocol. mAbs were analyzed in triplicate, while the ADC was analyzed once.

mAbs and the ADC mimic were analyzed in their formulation buffers, except for bevacizumab which was buffer-exchanged to BalanCD media and adjusted to a concentration of 1 mg/mL using 10 kDa molecular weight cutoff spin filters for initial method development and validation. To determine the limit of detection (LOD) and the limit of quantitation (LOQ) using a standard curve, concentrations were further adjusted for injection of protein amounts between 0.5 and 100 μg.

Bioreactor samples were obtained from a 10 day culture study using an anti-IL8–IgG1 producing CHO DP-12 cell line. Cells were grown in a batch 3 L culture using Applikon glass vessels. Cells were grown in BalanCD CHO Growth A media supplemented with 4.0 mM l-glutamine. Control samples were grown at a pH of 7.00 ± 0.05, a DO content of 40% of air saturation and a temperature of 37 °C. For stressed conditions, the value of each condition was changed after 6 days of culture. Low temperature samples were obtained by lowering the temperature to 32 °C, low DO samples were obtained by reducing the level of DO to 20%. Low temperature and low DO samples were obtained by simultaneously lowering both parameters to the levels previously outlined. Samples were taken on days 8 and 10 and were clarified by centrifugation at 1000*g* for 10 min and filtered through 0.45 and 0.20 μm PVDF membrane filters. Cell viability was measured using trypan blue exclusion and the levels recorded on the days of sampling can be found in Supporting Information, Table S1. Protein concentrations were determined *via* NanoDrop measurements and were between 0.06 and 0.16 mg/mL. The total manual preparation time of protein samples for analysis after sampling was between 12 and 13 min; however, this could be significantly reduced using online sampling systems with cell removal capabilities.

For binding studies, protein A was resuspended in 1× PBS and mixed with the antibody bevacizumab at a 1:1 molar ratio. The protein A–IgG mixture was mixed by pipette aspiration for approximately 1 min prior to injection.

### Protein A Chromatography–Mass Spectrometry

All analyses were performed using a Thermo Scientific Vanquish Flex Binary UHPLC system (Thermo Fisher Scientific, Germering, Germany) coupled online to a Thermo Scientific Ultra High Mass Range Q Exactive Plus hybrid quadrupole-Orbitrap mass spectrometer using an IonMax source with a HESI-II probe and a high-flow 32 gauge needle (P/N: 7005-60155) (Thermo Fisher Scientific, Bremen, Germany).

For mobile phase comparison experiments, two different mobile phase systems were employed. Mobile phase A1 was 50 mM aqueous ammonium acetate, pH 7.0, and mobile phase B1 was water, adjusted to pH 3.0 using LC–MS-grade acetic acid. Mobile phase A2 was 50 mM aqueous ammonium formate, pH 7.0, and mobile phase B2 was water, adjusted to pH 3.0 using LC–MS-grade formic acid. A MAbPac protein A column (4 × 35 mm, particle size 12 μm) was used for protein A affinity chromatography. The column temperature was maintained at 25 °C.

Buffer comparison was carried out using a flow rate of 0.500 mL/min, the gradient started with 0% B for 2 min followed by a step change to 100% B in 0.1 min which was held from 2.1 to 6 min. Re-equilibration to 0% B took place from min 6 until the end of the method at 8 min. During the first 2 min of the run the flow was diverted to waste using a six-port external valve on the instrument before redirection of the flow to the MS system. The UV acquisition wavelength was 280 nm.

All subsequent analyses were carried out using a refined setup relying on buffers A1 and B1; however, the pH of buffer B1 was reduced to pH 2.5. The flow rate was 0.500 mL/min and the gradient started with 0% B for 1.5 min followed by 100% B from 1.6 to 3.5 min. Re-equilibration to 0% B took place from 3.6 mins until the end of the method at 5 min.

Full MS spectra were acquired in positive polarity in a scan range of 2,000–15,000 *m*/*z*. The resolution was set to 25,000 at *m*/*z* 400, with an AGC target of 3 × 10^6^ ions and 10 microscans were performed. The maximum injection time was 200 ms. In-source trapping desolvation was set to −80 V, this was increased to −10 V for ADC analysis and the trapping gas pressure was set to 7.0. Detector *m*/*z* optimization was set to low *m*/*z*, while the ion transfer target *m*/*z* was set to high *m*/*z*. Sheath gas was set to 40 arbitrary units (AU) and auxiliary gas was set to 20 AU. The spray voltage was 3.8 kV, the capillary temperature was 320 °C, the S-lens RF was set to 200 V and the auxiliary gas heater temperature was 275 °C.

### Size Exclusion Chromatography–Mass Spectrometry

All analyses were performed using the same instruments as previously mentioned. A MAbPac SEC-1 (4 × 300 mm, particle size 5 μm, 300 Å) column was used for analysis and was maintained at 30 °C. All analyses were performed under isocratic flow conditions at 0.300 mL/min for 15 min. The buffer used was 50 mM LC–MS grade ammonium acetate. The pH was adjusted using LC–MS grade acetic acid until the target pH was reached. Full MS spectra were acquired in positive polarity in a scan range of 2,000–15,000 *m*/*z*. The resolution was 6,250 at *m*/*z* 400, with an AGC target of 3 × 10^6^ ions and 10 microscans were performed. A maximum injection time of 200 ms was used. In-source trapping desolvation was set to −150 V and trapping gas was set to 7.0. Detector *m*/*z* optimization was set to low *m*/*z*, while ion transfer target *m*/*z* was set to high *m*/*z*. Sheath gas was set to 30 AU and auxiliary gas was set to 15 AU. The spray voltage was 3.8 kV, the capillary temperature was 320 °C, S-lens RF was set to 200 V and the auxiliary gas heater temperature was 250 °C.

### Data Analysis

Mass spectra were acquired using Thermo Scientific Xcalibur version 4.1.31.9. The analysis of the acquired mass spectra was carried out using the Thermo Scientific BioPharma Finder software version 4.1. All software parameters used for data analysis are highlighted in Table S2. The raw data files were acquired in Xcalibur and data visualization was carried out using Thermo Scientific Chromeleon version 7.2.10.

## Results and Discussion

### Protein A Chromatography–MS Method Development

**Figure 1:**
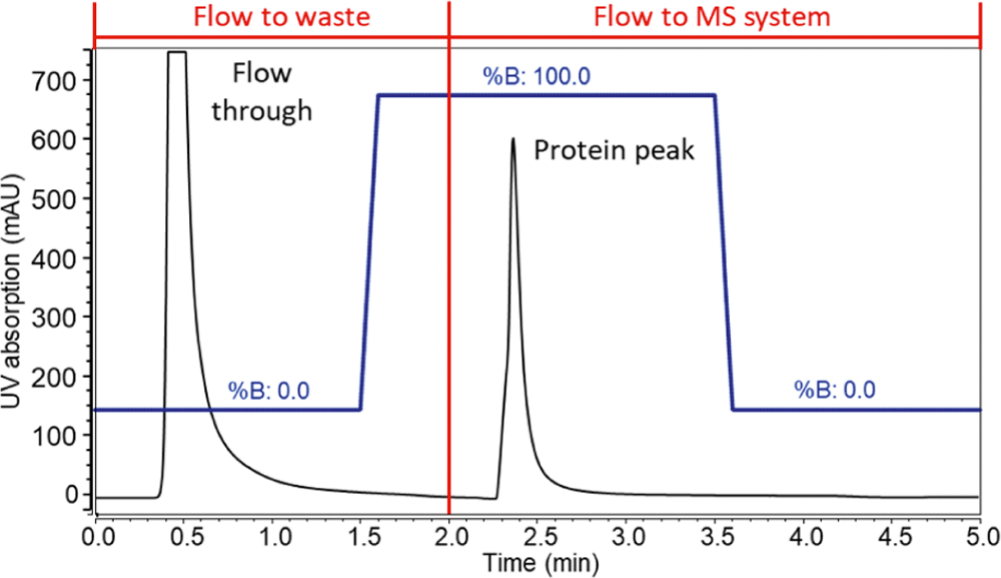
Optimized protein A chromatography method using 50 mM ammonium acetate, pH 7.0, as buffer A and acetic acid, pH 2.5, as buffer B. The black trace represents the chromatogram acquired *via* UV detection at 280 nm, while the blue trace represents the gradient applied. The first 2 min of the gradient the flow was diverted to waste, as indicated in red, and a flow rate of 0.500 mL/min was used.

The development of the protein A-MS (ProA-MS) protocol began by investigating two different aqueous MS-friendly mobile phase systems which were based on ammonium acetate and acetic acid or ammonium formate and formic acid. Bevacizumab drug product (25 μg) was injected on column and UV and MS detection was performed. The results from both approaches were compared and can be seen in Figure S1. Based on UV absorption, both buffer systems show a similar peak profile and comparable elution time, peak width and symmetry. Nevertheless, MS signal intensity and the signal to noise ratio (S/N) of the raw spectra acquired were found to be superior using ammonium acetate. With the main charge states being in the range of +23 to +29, both charge envelopes clearly infer a native-like protein conformation. The spectral profile acquired shows a slight shift toward higher *m*/*z* values in the case of the ammonium acetate mobile phases when compared to ammonium formate, indicating that the protein higher order structure is seemingly better preserved with ammonium acetate. Similar findings were reported in the past and are in accordance with ammonium acetate being a kosmotropic salt, stabilizing protein structures, while ammonium formate is chaotropic.^12^ The findings from this initial study indicated that a mobile phase based on ammonium acetate was superior for use along with ProA-MS. Subsequently, a comparison of this mobile phase system with PBS, a conventional MS-incompatible mobile phase was undertaken, which can be seen in Figure S2. The peak asymmetry was 1.44 for the nonvolatile buffers, while it was 1.59 in case of the volatile buffers. The peak width at half height also increased from 0.056 to 0.067 when moving from the nonvolatile to volatile mobile phase system. This indicates that the chromatographic performance marginally declines when volatile mobile phases are employed. The chromatographic gradient and method duration were next optimized using bevacizumab in cell culture media. The aim was to optimize the gradient and to reduce the overall run time to as low as possible while maintaining enough time to ensure full protein binding, removal of all cell culture contaminants, full protein elution and sufficient equilibration back to starting conditions. Figure 1 shows the final method with a run time of 5 min composed of 1.5 min at 100% A for protein loading, 2 min elution at 100% B and 1.5 min of re-equilibration again at 100% A. The first 2 min the flow was diverted to waste to ensure full removal of all unbound cell culture contaminants, which could interfere with MS detection. As can be seen in Figure S3, using a pH of 3.0 for the elution buffer resulted in a wider peak compared to pH 2.5. In addition, it was found that a lower mobile phase B pH yields a 17.9% better recovery compared to the mobile phase of pH 3.0. For this reason, all subsequent analyses were carried out at pH 2.5.

A flow rate of 0.5 mL/min required careful selection of gas and temperature parameters in the MS ion source to ensure full molecular desolvation and protein ion declustering. MS parameters, including the MS resolution setting, were optimized to achieve maximum MS signal response. The resolution setting used must be optimized to balance sensitivity while also resolving isoforms with similar masses in order to ensure acceptable mass accuracy.^14^ This is particularly important when near-isobaric variants are not chromatographically separated before detection.^24^

**Figure 2:**
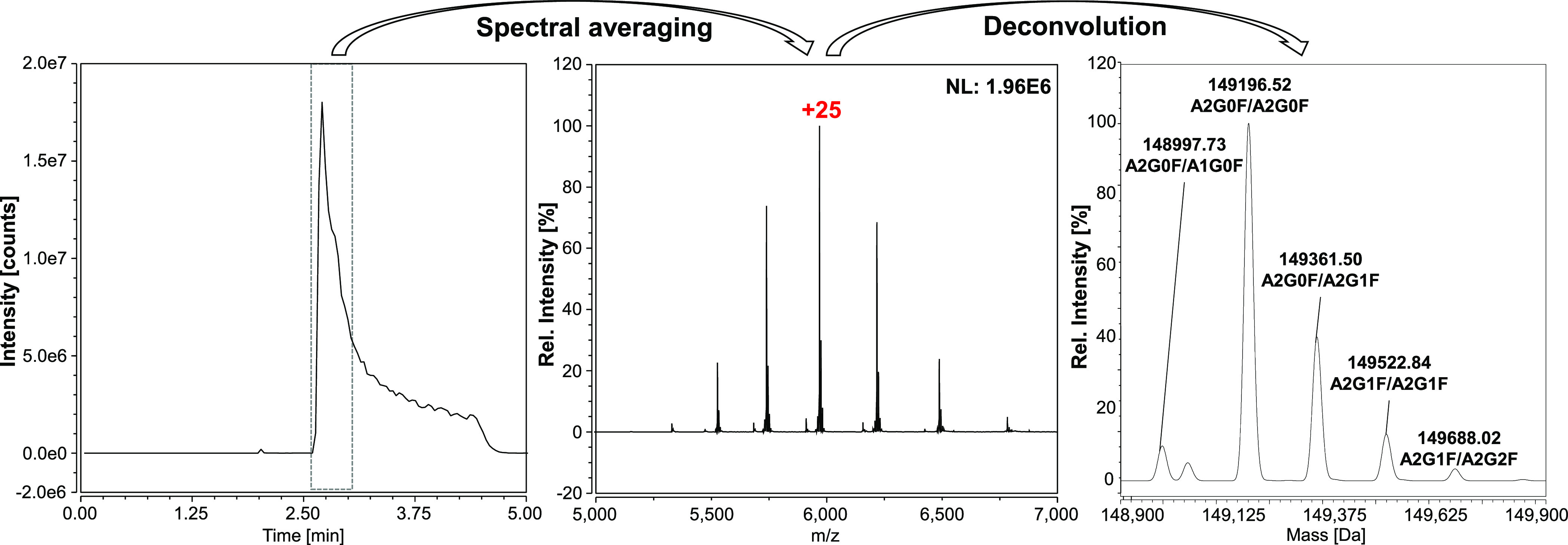
Raw data and deconvoluted spectrum acquired through injection of 25 μg of bevacizumab using the ProA-MS method. The left panel shows the TIC trace and the middle panel displays the protein charge envelope obtained through spectral averaging of the observed peak, the most abundant charge state is highlighted. The right panel shows the deconvoluted spectrum with annotated glycoforms.

Figure 2 shows the TIC chromatogram of 25 μg of bevacizumab along with the mass spectra obtained from averaging of the TIC peak and the annotated deconvoluted spectra. Spectral averaging of the main peak shows a native-like charge envelope for bevacizumab without any traces of adduction. Deconvolution of the charge envelope shows four distinct peaks which correspond to the main bevacizumab glycoforms with A2G0F/A2G0F being the most abundant followed by higher galactosylated forms. The average mass accuracy across three runs for the annotated glycoforms was within 30 ppm. The charge envelope and deconvoluted spectra obtained using the ProA-MS method correspond well to those previously reported for bevacizumab.^14,25^

Method precision was tested by investigating the relative standard deviation of the retention time and relative peak area acquired using six replicate injections of bevacizumab based on UV detection. The relative standard deviation for the retention time was <0.001%, indicating extremely high retention time precision across all runs, while the relative standard deviation for the peak area was 0.321%, also indicating high levels of precision. Method robustness with regard to temperature and mobile phase pH was tested through triplicate injections of bevacizumab at an increased column temperature (30 °C) and under application of a mobile phase B with increased pH (pH 2.8). At higher temperature, the relative standard deviation for the retention time and peak area was 0.31 and 1.32%, respectively. At higher mobile phase pH, these values were 1.63 and 2.41%, respectively. This indicates that the method is robust and that the mobile phase pH has a larger impact on elution time and peak area compared to temperature.

Method linearity was investigated by diluting bevacizumab in chemically defined cell culture media to a concentration of 1 mg/mL and injecting between 0.5 and 100 μg of the material. By plotting the UV peak area against the amount of mAb injected, a linear trend was established as expected, with an *R*^2^ value of 0.9993, Figure S4A. The LOD based on UV detection was calculated to be 3.7 μg, Table S3. The LOQ was calculated to be 11.25 μg; however, Figure S4B shows that the mass spectral quality obtained using as little as 0.5 μg of mAb is comparable to the mass spectra obtained using 100 μg of mAb. The charge envelopes obtained for both injection amounts are highly similar and deconvolution of the mass spectra allowed for annotation of the three main glycoforms with a mass deviation of less than 30 ppm in either case. In addition, the method also allows for titer determination down to the specified LOQ.

### Application to Monoclonal Antibodies of Varying Complexity

**Figure 3:**
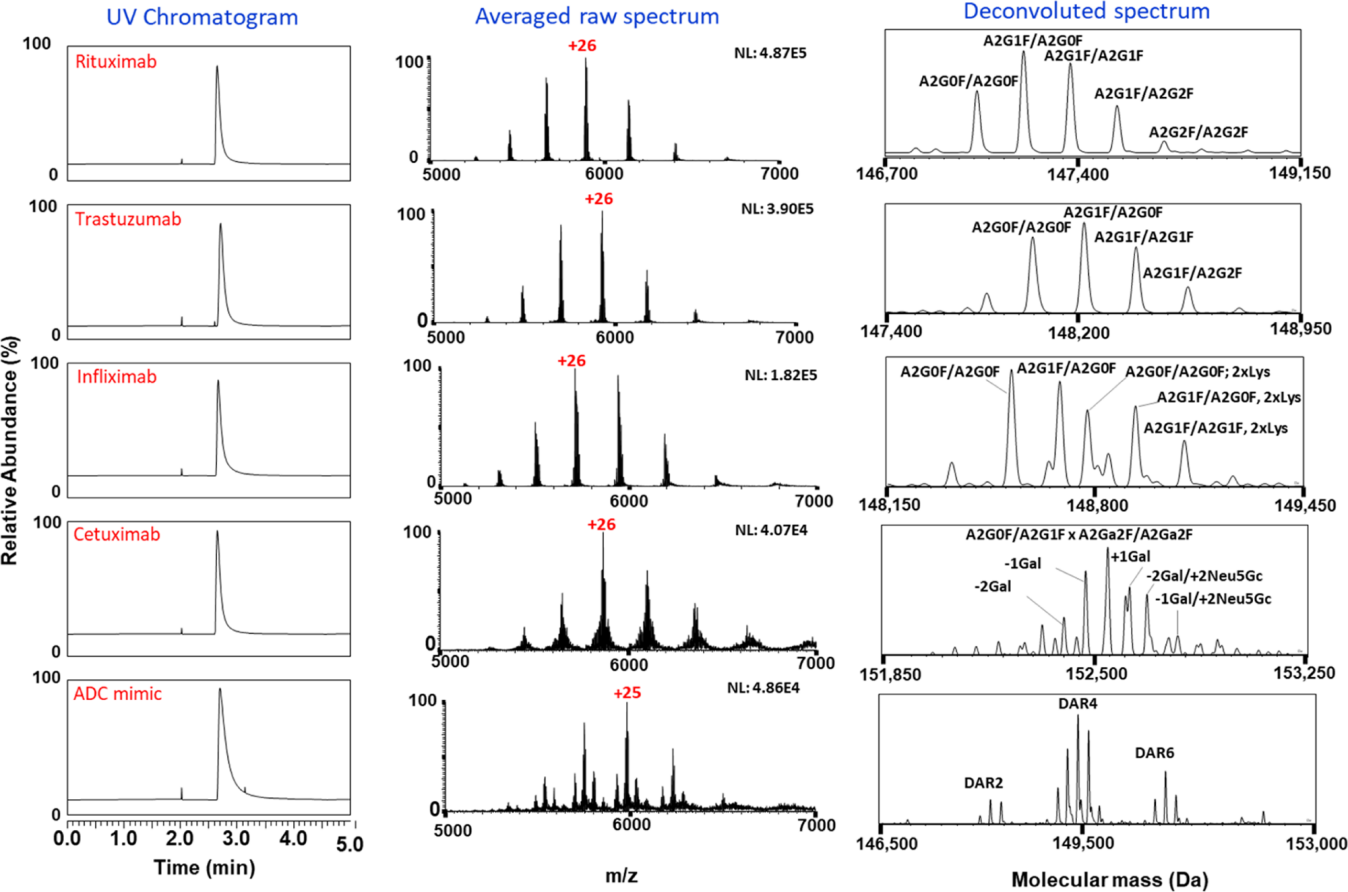
ProA-MS was applied to a number of IgG1 mAbs of varying complexity, namely, rituximab, trastuzumab, infliximab, cetuximab and an ADC mimic. The UV profile of each biotherapeutic can be seen in the left panel, while the middle panel shows the averaged raw spectrum obtained through integration of the protein peak. The annotation of the peaks from deconvoluted spectra is shown in the right panel.

ProA-MS was applied to a number of biotherapeutics that varied in structural complexity. These were rituximab, trastuzumab, infliximab, cetuximab and an ADC mimic, the resulting data are presented in Figure 3. UV acquisition showed no distinct differences between samples except for a slightly higher degree of peak tailing for the ADC mimic when compared to the mAbs. Spectral averaging of the peaks again revealed a native-like protein charge envelope between 5,000 and 7,000 *m*/*z* in each case, however with clearly varying degrees of complexity.

Rituximab and trastuzumab were observed to be less complex compared to the other samples analyzed and have been well characterized in previous studies.^9,14,26^ The most abundant glycoforms detected for both molecules were A2G1F/A2G0F, while the second most abundant glycoform differed, A2G1F/A2G1F for rituximab and A2G0F/A2G0F for trastuzumab. These results correspond well with those previously reported.^9^ Infliximab annotation is more complicated as the molecule exhibits highly abundant charge variants derived from incomplete C-terminal lysine truncation due to low carboxypeptidase activity.^27^ This modification results in a mass difference of ∼128 Da which, to some degree depending on the MS resolution, can cause an overlap with glycoforms with one additional galactose and thus, potentially results in compromised annotation. Using the ProA-MS method, it was found that most abundant species corresponded to A2G0F/A2G0F, A2G0F/A2G1F and A2G1F/A2G1F glycoforms of infliximab with varying degree of C-terminal lysine. The annotation of cetuximab isoforms is highly challenging due to an additional glycosylation site in the Fab region. The charge envelopes and deconvoluted spectral profiles acquired using ProA-MS, however, corresponded well with those previously acquired using cation-exchange chromatography with pH gradient elution coupled to native MS (CEX–MS).^17^ The main cetuximab variant is represented by the Fc and Fab glycan pairs A2G0F/A2G0F and A2Ga2F/A2Ga2F. Still highly prominent but less abundant forms are caused by various degrees of galactosylation and sialylation with the sialic acids being of the *N*-glycolylneuraminic acid type, again correlating with findings of previous studies.^17,28,29^

Traditional ADCs are produced through a series of reactions on lysine side chains or on cysteine thiols following reduction of interchain disulfide bonds, which results in heterogeneous mixtures of DARs.^30^ The average DAR is an important critical quality attribute (CQA) that can affect the safety and efficacy of an ADC.^31^ Moreover, ADCs which are based on a monoclonal antibody scaffold exhibit other layers of complexity typical for mAbs, such as differential glycosylation. In general, two layers of complexity were identified upon ProA-MS, different DARs and varying glycosylation. The DAR forms found were DAR 2, 4 and 6 and glycoform pairings found were A2G0F/A2G0F, A2G1F/A2G0F, A2G1F/A2G1F and A2G1F/A2G2F. The average DAR was calculated from the ProA-MS spectral data to be 3.7. Importantly, this ADC mimic is a cysteine-conjugated ADC mimic. The fact that an intact protein charge envelope can be observed and can be used for annotation and quantitation demonstrates applicability of the presented method for rapid analysis of protein complexes containing antibodies with exposed Fc domains and formed through noncovalent bonds. Notably, the ADC shows a higher level of tailing compared to the mAbs. This could potentially be caused by the presence of hydrophobic payloads; however, no clear correlation between the drug loading level and elution within the protein A peak was found. A wider *m/z* range (1,000-12,000) is presented in Figure S5, which shows that the degree of smaller *m/z* species, which could potentially be due to method induced dissociation, is negligibly small, supporting the claim of ProA-MS being a very gentle analysis method. Calculated mass deviations were low in a majority of cases. In some instances, higher mass deviations had to be accepted as some MS peaks are inevitably composed of different near-isobaric protein isoforms and the reported masses will not represent a single species but rather the average of several species. Nevertheless, in such cases, average mass deviations calculated did still not exceed 30 ppm. Details on all reported isoforms are presented in Table S4, Supporting Information.

### Analysis of mAbs Expressed in Bioreactors Operated under Differential Culture Conditions

**Figure 4:**
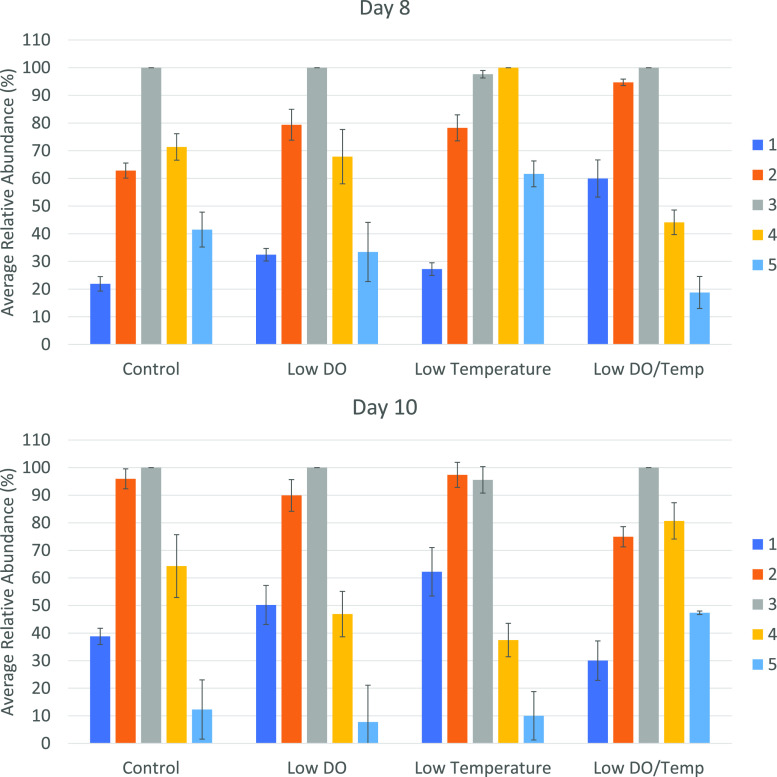
Average relative abundance of the main monoclonal antibody isoforms detected using protein A-MS on samples taken from bioreactors that were grown under altered conditions. Different colors represent different isoforms and the error bars represent the variability based on triplicate injections.

As previously mentioned, biotherapeutics are produced using animal cell culture technologies. Production can happen at various scales and the parameters used during cell culture can affect the CQAs of the product. For example, decreasing the temperature of the culture can increase the production titer and has also been shown to reduce sialidase activity.^32^ DO levels were also found to impact the glycoprofile of biotherapeutics with reduction of galactosylation reported when the levels of DO were reduced.^33,34^ The effect of these altered parameters, low temperature, low DO and a combination thereof, was investigated using the established ProA-MS method. Samples were grown under altered bioprocessing conditions along with a control sample to examine the effect of the environmental changes on the product quality attribute (PQA) profile of the mAb. Conditions were altered on day 6 of the cell culture and samples were taken on days 8 and 10, resulting in an exposure time of either 2 or 4 days. Triplicate ProA-MS injections of 4 μg each were performed to evaluate changes in the PQA profile reflected by relative MS signal abundances of the protein isoforms seen, Figure 4.

Previous characterization through peptide mapping analysis has revealed the typical differential N-glycosylation and an N-terminal “VHS” sequence tag which amounts to an additional 323 Da being the main sources of heterogeneity on this mAb (data not shown).^35^ The lowest molecular weight variant depicted in Figure 4 (form 1) corresponds to an antibody with no N-terminal modification and an A2G0F/A2G1F glycan pair. All other variants show a successive mass increase of 162 Da, from left to right, caused by the above described modifications. For simplicity sake these isoforms are referred to as forms 1 to 5. A putative identification of isoforms and information on experimental and theoretical masses and mass deviations are provided in Table S5 of the Supporting Information.

The low DO sample of day 8 appears to show a shift in the isoform pattern toward a slightly higher abundance of lower molecular weight forms. The same trend but more pronounced is visible upon low DO and low temperature exposure, while the opposite is the case for the samples only exposed to low temperature. Changes are significant with form 5 showing relative abundances elevated by almost 30% when compared to the control sample. A different trend is visible after cultivation for 10 days. In contrast to day 8, the control samples of day 10 appear to be of lower molecular weight with form 2 being almost as abundant as form 3. Exposure to low DO and low temperature separately is visibly leading to an increase in the lower molecular weight form 1, while forms 4 and 5 slightly decrease. A reversed trend is visible when both altered bioprocessing parameters are combined with forms 4 and 5 showing a clearly elevated level, whereas form 1 has decreased to below a control sample level. In summary, changes in DO and temperature can have a profound impact on the monoclonal antibody quality profile, in particular, on antibody glycan galactosylation. Also, changes seem to strongly depend on the time of cultivation as different trends were observed for day 8 and 10 samples.

The bioreactor study showed that ProA-MS is a valuable tool to distinguish different product profiles of monoclonal antibody cell cultures and can provide rapid information on the effect various parameter changes and feeding strategies can have on PQAs with a high level of mass accuracy (<30 ppm, Table S5).

### SEC–MS to Probe Stoichiometry of Protein A–IgG Binding

**Figure 5:**
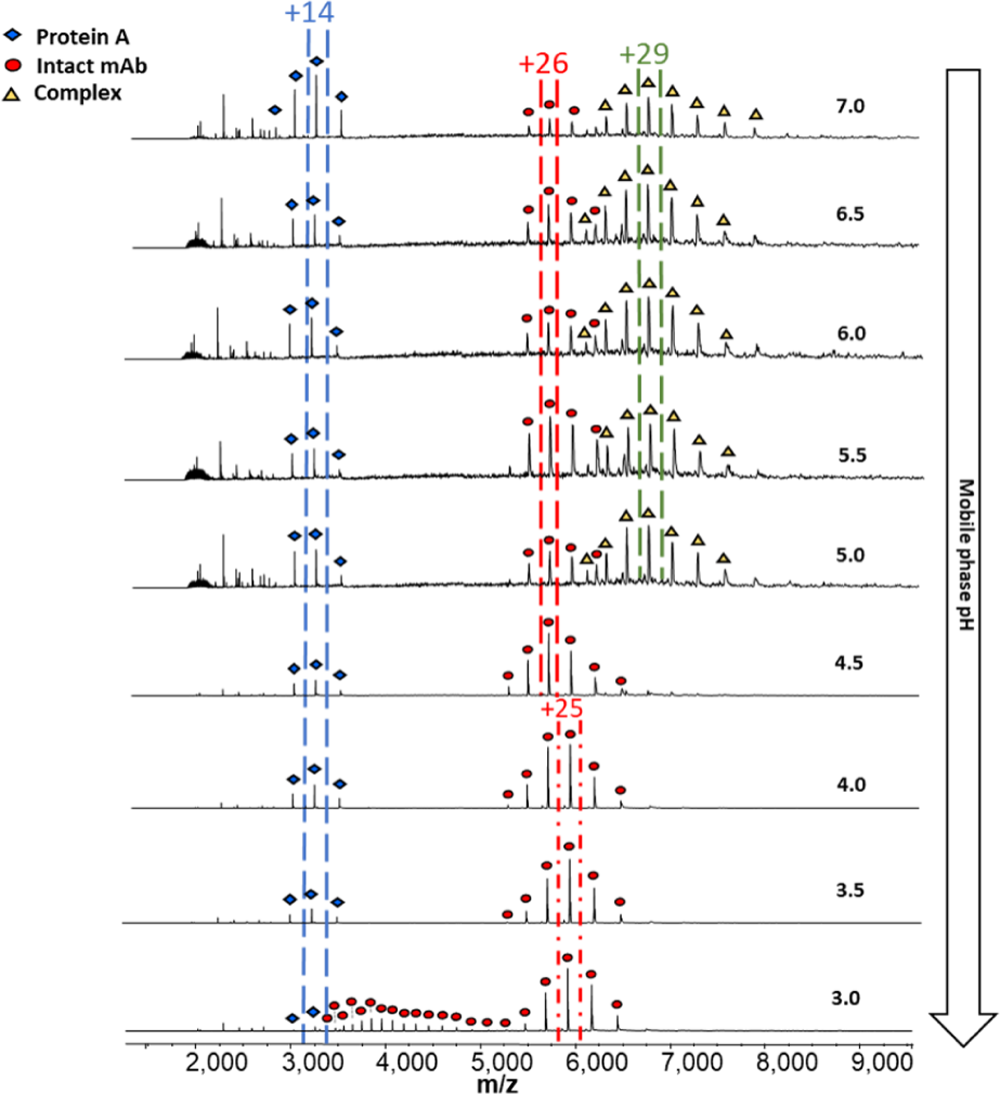
MS spectra showing the effect of pH on protein A, IgG and the complex thereof. Blue squares indicate charge states that belong to the protein A molecule, red dots symbolize charge states from unbound IgG and yellow triangles mark charge states which belong to a complex thereof at a 1:1 molar ratio. The most abundant charge states are highlighted and their presence across the pH ranges is mapped out.

Using nondenaturing SEC–MS, it was attempted to further understand the pH dependency of structures of mAb and protein A and of their formed complex. For this study, soluble protein A was resuspended in 1× PBS and bevacizumab was diluted to 1 mg/mL in 1× PBS. As previously reported, the molar binding ratio for mAb and protein A is in the range of 1:1 to 1:2. For this experiment, protein A and the mAb were mixed in a molar ratio of 1:1 as results showed complex formation, while unbound protein A and unbound mAb were also still observable. Upon analysis, it was evident that the dominant species were unbound protein A, unbound mAb, and a complex with a stoichiometry of 1:1, while no larger complexes were observed. Subsequently, SEC–MS analysis was performed at different mobile phase pH values ranging from pH 7.0 to 3.0 in 0.5 pH unit steps. MS spectra across the chromatographic elution range are shown in Figure 5.

The result shows that, as expected, when the pH is lowered to more acidic conditions, the protein A–mAb complex dissociates. This dissociation begins to occur at pH 4.5 and happens to an extent where no complex is visible any longer. The spectra at increasingly lower pH show that at pH 3.0, the mAb begins to partially denature or undergo a structural change, which is clearly indicated by an extension of the charge envelope to a lower *m*/*z* region. Comparing this to the results obtained from the ProA-MS experiments, there is a discrepancy as a pH of 2.5 during ProA-MS did not result in any MS inferred denaturation of the protein. The most likely explanation is that protein elution in protein A chromatography is faster than the kinetics of denaturation, while in SEC–MS, the protein is exposed to acidic conditions for a much more extended period of time due to the longer chromatographic run times involved. Also, there is the possibility that the column is buffering against a change to pH 2.5 and that the true pH on the column is slightly higher which would favor preservation of a native-like structure.

## Conclusions

Protein A chromatography remains a frontline technique for the capture and purification of monoclonal antibodies from cell culture supernatant. This study described the adaptation of this purification technique to a quick and powerful analysis strategy using MS-friendly mobile phases and through direct interfacing to native MS. Five minutes was found to be enough time to ensure full protein binding, removal of contaminants, elution and column re-equilibration to starting conditions while maintaining reproducibility and robustness. The MS data quality observed was excellent and allowed for the analysis of low abundant glycoforms of a monoclonal antibody with as little as 0.5 μg of material consumed. The method has proven generic applicability through the analysis of multiple Fc region-bearing biopharmaceuticals including complex proteins such as cetuximab and an ADC mimic. The LC and MS conditions were chosen clearly to favor the preservation of the native protein structure and noncovalent bonds, making therapeutic formats such as cysteine-conjugated ADCs also amenable for analysis. ProA-MS was further employed to investigate the effect of altered culture parameters on the quality profiles of samples acquired from a number of bioreactors and allowed for the detection of differential glycosylation based on these changes. Next to analytical techniques such as SEC–MS, IEX–MS, or HIC–MS, this method represents yet another potent tool in the toolbox of native LC–MS analysis strategies for biopharmaceuticals. A simple analysis setup, quick run times and high sensitivity paired with the capabilities to analyze proteins directly in media render the presented method a highly promising tool for PAT applications for a variety of biopharmaceutical products.

In addition, native SEC–MS was applied to further understand the effect of pH on the molecular binding of protein A and monoclonal antibodies. A complex was observed composed of a single molecule each, protein A and a mAb, which was found to be stable down to pH 4.5. Moreover, it was shown that protein denaturation starts at a pH of 3.0 and that denaturation appears to be a gradual rather than an instant process as no trace of denaturation was observed at even lower pH upon protein A elution.

## Supporting Information Available

Chromatograms outlining ProA-MS buffer development, standard curves and quantitation data, parameters used for deconvolution of native mass spectra, and experimental mass data for the various molecules analyzed (PDF)
