# Supplementary material for: Rapid Analysis of Biotherapeutics Using Protein A Chromatography Coupled to Orbitrap Mass Spectrometry
Source: Anal Chem. 2021 Sep 29;93(40):13505–12. doi: 10.1021/acs.analchem.1c02365 (PMC8515350; doi:10.1021/acs.analchem.1c02365)
Supplement: Supplementary file 1 — ac1c02365_si_001.pdf [file ac1c02365_si_001.pdf]

Supporting Information

Rapid Analysis of Biotherapeutics Using Protein A Chromatography Coupled to Orbitrap Mass Spectrometry

Craig Jakes[1,2], Florian Füssl[1], Izabela Zaborowska[1] and Jonathan Bones[1,2], *

[1]Characterisation and Comparability Laboratory, The National Institute for Bioprocessing Research and Training, Fosters Avenue, Mount Merrion, Co. Dublin A94 X099, Ireland

[2]School of Chemical and Bioprocess Engineering, University College Dublin, Belfield, Dublin 4, D04 V1W8, Ireland

*To whom correspondence should be sent, tel: +353 1215 2100, fax: +353 1215 8116, email: jonathan.bones@nibrt.ie

Index of Figures and Tables

Figure S1: The UV chromatograms (left) and the average spectral profiles (right) of Bevacizumab acquired using ProA-MS with buffer system 1 (ammonium acetate) and buffer system 2 (ammonium formate). ---3
Figure S2: The UV chromatogram profile of 25 µg of Bevacizumab obtained using a non-volatile buffer system (left) compared to a volatile buffer system (right). Peak width and asymmetry values obtained for each buffer system are highlighted below each profile. ---4
Figure S3: The UV profile of Bevacizumab using an elution buffer of pH 3.0.--------------------------------5
Figure S4: **(A)** A standard curve of protein concentration ranging from 0.5 to 100 µg of Bevacizumab in media was established to determine the LOD and LOQ of the protein A mass spectrometry method. **(B)** The corresponding raw (mid panel) and deconvoluted spectra (right panel) show that confident annotation is still possible using as little as 0.5 µg of sample.--------------------------------------6
Figure S5: Mass spectral profile of 10µg of ADC acquired between 1,000-12,000 m/z using protein A mass spectrometry.--7

Table S1: Bioreactor cell viability details from cell culture samples of days 8 and 10. 8
Table S2: Parameters used for deconvolution of native mass spectra using Biopharma Finder 4.1. 9
Table S3: Calculation of LOD and LOQ. A standard curve was used for determination of linearity of signal response, LOD and LOQ based on the UV signal of the main peak. 10
Table S4: Average experimental mass and the calculated average mass deviation for the glycoforms from various mAbs and ADC analysed through ProA-MS. 11
Table S5: Average experimental mass (n=3 unless indicated otherwise) and the calculated average mass deviation for the glycoforms putatively identified from the bioreactor study analysed through ProA-MS. “VHS” indicates the presence of a “VHS” amino acid sequence tag as a N-terminal modification. 13

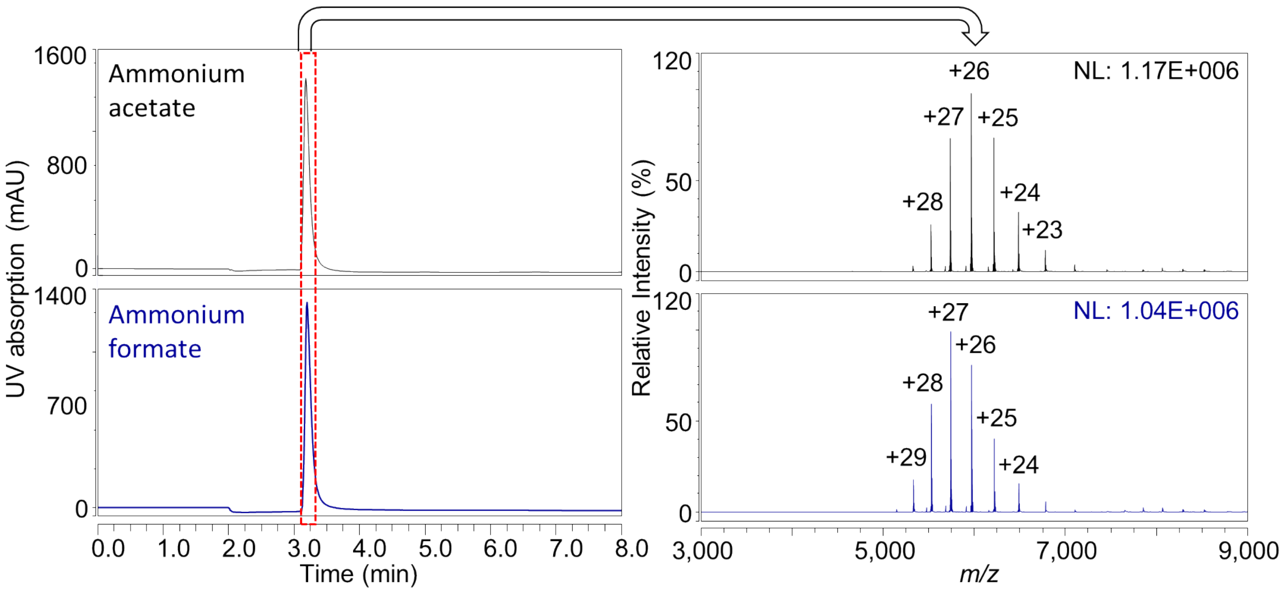

Figure S1: The UV chromatograms (left) and the average spectral profiles (right) of Bevacizumab acquired using ProA-MS with buffer system 1 (ammonium acetate) and buffer system 2 (ammonium formate).

A: PBS @ pH 7.0
B: PBS @ pH 2.5

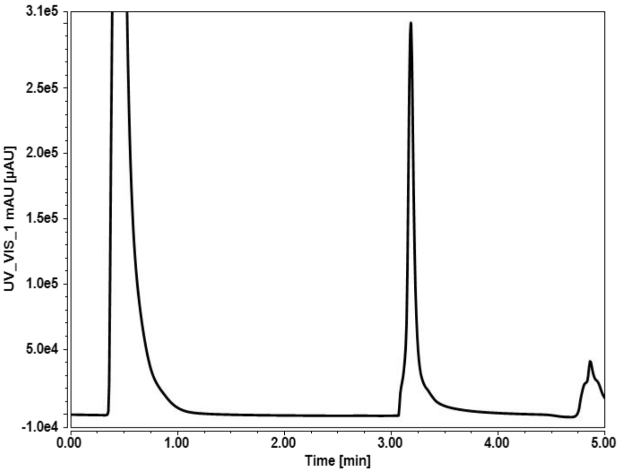

Peak Width (50%): 0.056
Asymmetry: 1.44

A: 50mM Ammonium Acetate @ pH 7.0
B: Water adjusted to pH 2.5 with Acetic Acid

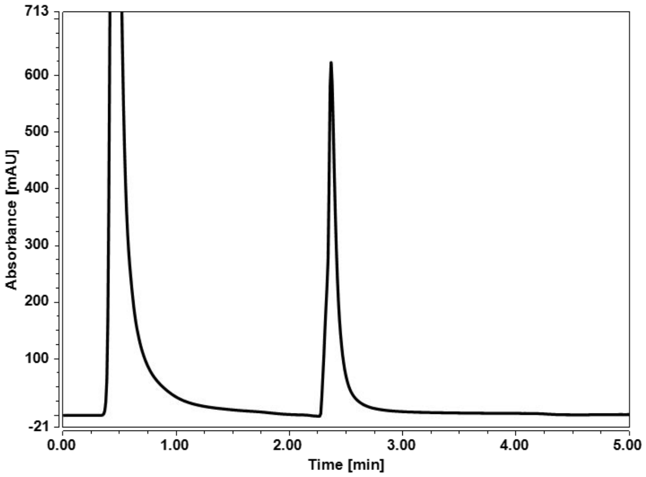

Peak Width (50%): 0.067
Asymmetry: 1.59

Figure S2: The UV chromatogram profile of 25 µg of Bevacizumab obtained using a non-volatile buffer system (left) compared to a volatile buffer system (right). Peak width and asymmetry values obtained for each buffer system are highlighted below each profile.

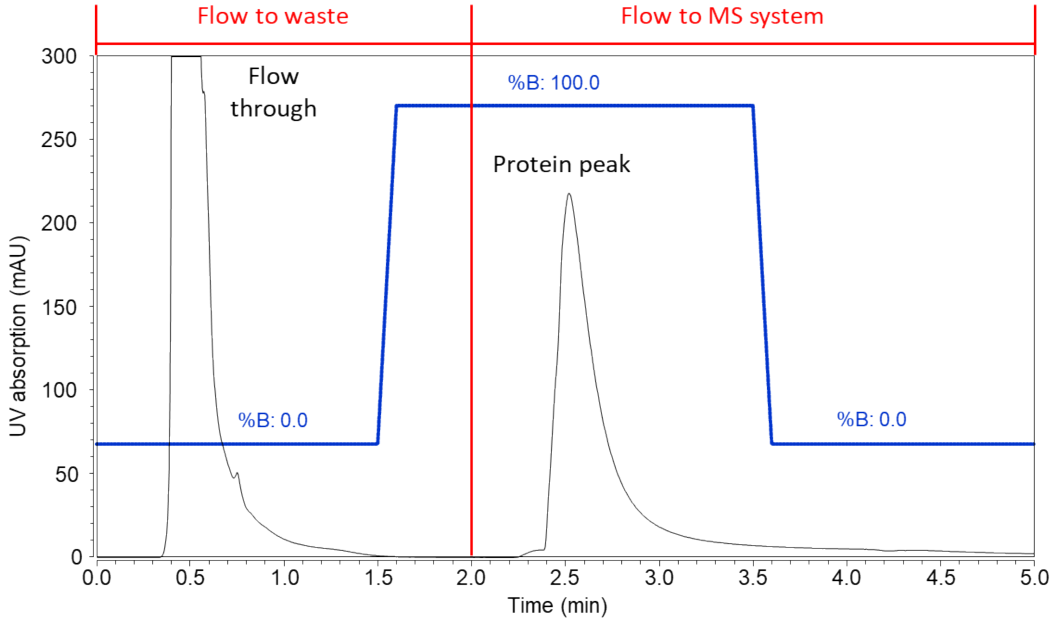

Figure S3: The UV profile of Bevacizumab using an elution buffer of pH 3.0.

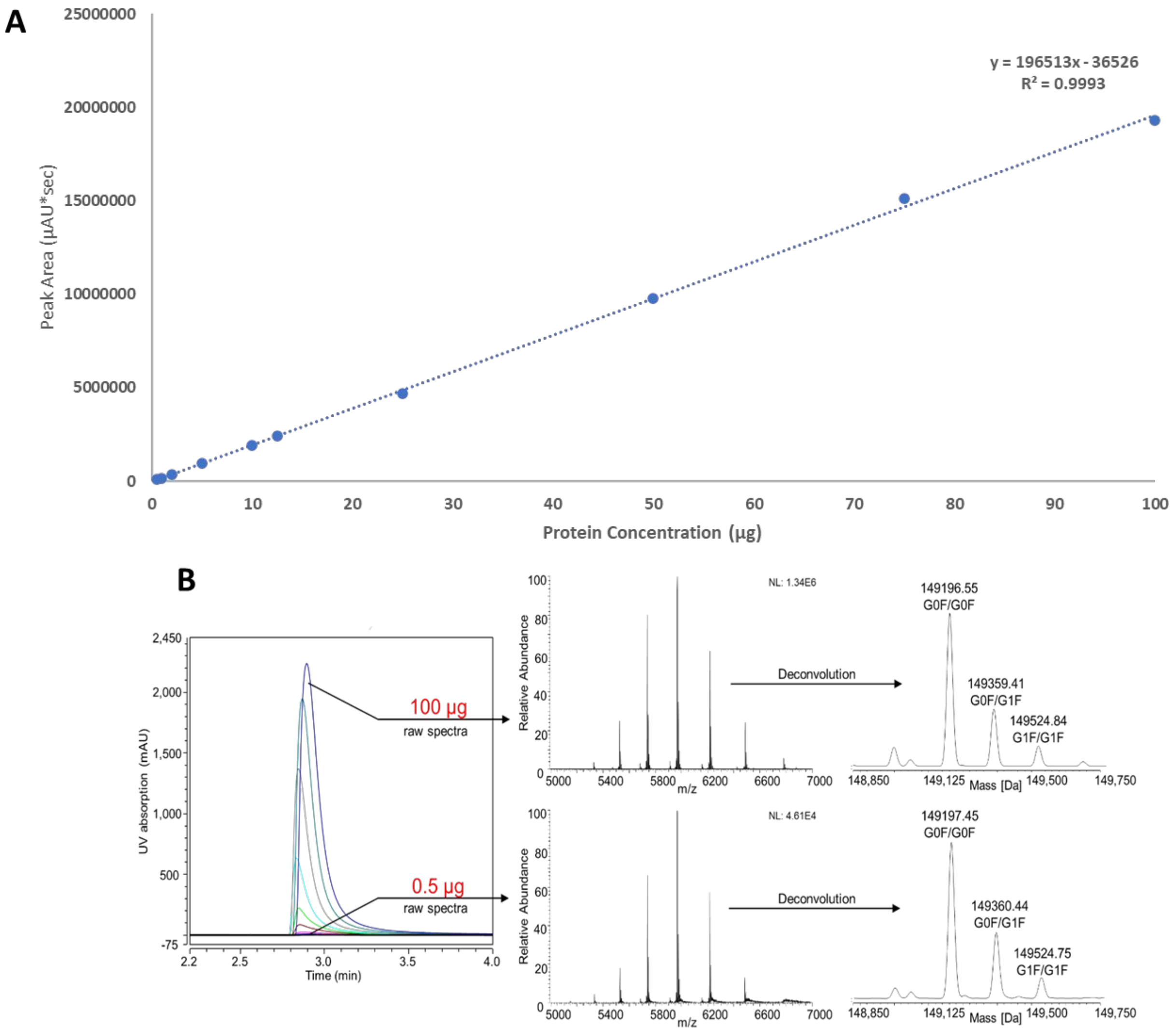

Figure S4: (A) A standard curve of protein concentration ranging from 0.5 to 100 µg of Bevacizumab in media was established to determine the LOD and LOQ of the protein A mass spectrometry method. **(B)** The corresponding raw (mid panel) and deconvoluted spectra (right panel) show that confident annotation is still possible using as little as 0.5 µg of sample.

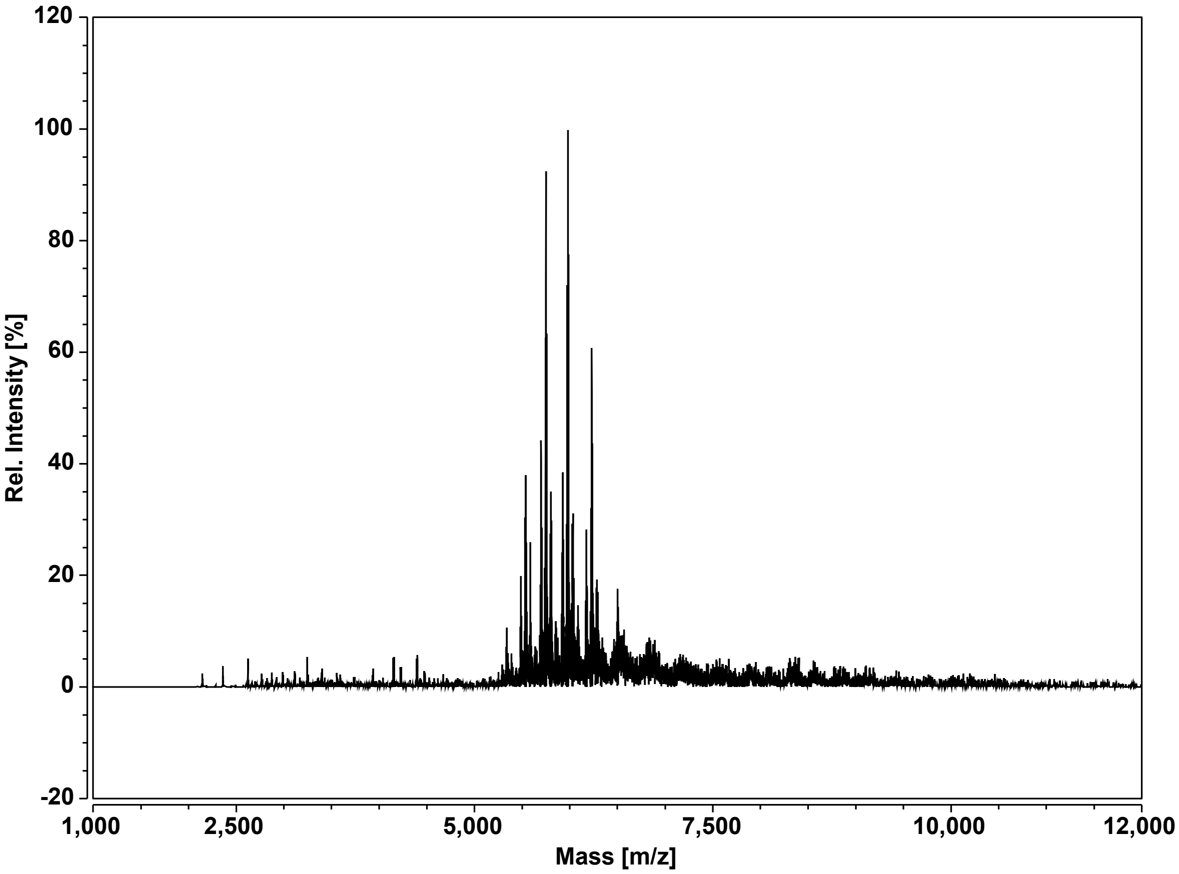

Figure S5: Mass spectral profile of 10µg of ADC acquired between 1,000-12,000 m/z using protein A mass spectrometry.

Table S1: Bioreactor cell viability details from cell culture samples of days 8 and 10.

Condition	Day 8	Day 10
**Control**	97%	78%
**Low DO**	91%	84%
**Low Temperature**	94%	90%
**Low DO and Temperature**	96%	89%

Table S2: Parameters used for deconvolution of native mass spectra using Biopharma Finder 4.1.

Source Spectra Method	Average Over Selected Retention Time
m/z Range	1,000.000 – 15,000.000
Chromatogram Trace Type	BPC
Deconvolution Algorithm	ReSpect
Output Mass Range	10,000 – 1,000,000
Charge State Range	5 – 100
Minimum Adjacent Charges	4 – 4
Target Mass	160,000.000 Da

Table S3: Calculation of LOD and LOQ. A standard curve was used for determination of linearity of signal response, LOD and LOQ based on the UV signal of the main peak.

**Protein amount injected (µg)**	**Peak Area (µAU*sec)**
100<br>100<br>100	18964573<br>19227124<br>19801586
75<br>75<br>75	15004905<br>15133676<br>15246857
50<br>50<br>50	9639377<br>9860918<br>9899179
25<br>25<br>25	4557786<br>4815190<br>4753895
12.5<br>12.5<br>12.5	2349821<br>2426830<br>2444999
10<br>10<br>10	1857888<br>1981184<br>1979389
5<br>5<br>5	907362<br>996547<br>971190
2<br>2<br>2	359864<br>368469<br>369354
1<br>1<br>1	121453<br>123885<br>174842
0.5<br>0.5<br>0.5	84215<br>79643<br>62280
**LOD/LOQ Calculations**	
**Slope (S)**	**196513**
**Standard Deviation of response (Sy)**	**221008.5**
**LOD (3.3(Sy/S))**	**3.71 µg**
**LOQ (10(Sy/S))**	**11.25 µg**

Table S4: Average experimental mass and the calculated average mass deviation for the glycoforms from various mAbs and ADC analysed through ProA-MS.

Glycoform	Theoretical Mass	Average Experimental Mass (n=3)	Average Mass Deviation (ppm) (n=3)
**Bevacizumab**			
**A2G0F/A1G0F**	148993.871	148997.730	25.90
**A2G0F/A2G0F**	149197.0687	149196.757	-2.09
**A2G0F/A2G1F**	149359.2095	149361.297	13.97
**A2G1F/A2G1F**	149521.3504	149524.447	20.70
**A2G1F/A2G2F**	149683.4912	149687.84	29.05
**Rituximab**			
**A2G0F/A2G0F**	147074.8571	147076.40	10.49
**A2G0F/A2G1F**	147236.998	147237.51	3.50
**A2G1F/A2G1F**	147399.1388	147399.97	5.64
**A2G1F/A2G2F**	147561.2797	147563.11	12.40
**A2G2F/A2G2F**	147723.4205	147725.95	17.12
**Trastuzumab**			
**A2G0F/A2G0F**	148056.0733	148058.75	18.08
**A2G0F/A2G1F**	148218.2141	148220.15	13.06
**A2G1F/A2G1F**	148380.355	148381.49	7.65
**A2G1F/A2G2F**	148542.4958	148545.52	20.38
**Infliximab**			
**A2G0F/A2G0F**	148512.043762	148514.1	13.91
**A2G0F/A2G1F**	148674.184610	148678.5	28.91
**A2G0F/A2G0F x 2 Lys**	148768.389762	148770.3	13.01
**A2G0F/A2G1F x 2 Lys**	148930.530610	148932.8	15.43
**A2G1F/A2G1F x 2 Lys**	149092.671457	149095.7	20.22
**Cetuximab**			
**A2G0F/A2G0F x A2Ga2F/A2Ga2F**	152514.0363	152518.4	28.3
**+1 Gal**	152676.1819	152678.6	15.9
**-1 Gal**	152351.8907	152352.7	5.2
**-2 Gal**	152189.7452	152191.6	12.3
**-2Gal/+2Neu5Gc**	152804.2635	152805.3	7.0
**-1Gal/+2Neu5Gc**	152966.4091	152968.3	12.5
**ADC**	**Theoretical Mass**	**Experimental Mass**	**Delta PPM**
**DAR2**			
**A2G0/A2G0F**	147856.5902	-	-
**A2G0F/A2G0F**	148002.7364	148000.88	-12.54
**A2G0F/A2G1F**	148164.8772	148163.05	-12.33
**A2G1F/A2G1F**	148327.0181	148327.53	3.45
**A2G1F/A2G2F**	148489.1589	-	-

**DAR4**			
**A2G0/A2G0F**	149192.5902	149192.13	-3.08
**A2G0F/A2G0F**	149338.7364	149341.50	18.51
**A2G0F/A2G1F**	149500.8772	149504.92	27.04
**A2G1F/A2G1F**	149663.0181	149664.22	8.03
**A2G1F/A2G2F**	149825.1589	149825.16	0.01
**DAR6**			
**A2G0/A2G0F**	150528.5902	-	-
**A2G0F/A2G0F**	150674.7364	150678.48	24.85
**A2G0F/A2G1F**	150836.8772	150840.47	23.82
**A2G1F/A2G1F**	150999.0181	151002.52	23.19
**A2G1F/A2G2F**	151161.1589	-	-
**DAR8**			
**A2G0/A2G0F**	151864.5902	-	-
**A2G0F/A2G0F**	152010.7364	-	-
**A2G0F/A2G1F**	152172.8772	-	-
**A2G1F/A2G1F**	152335.0181	152335.70	4.48
**A2G1F/A2G2F**	152497.1589	-	-

Table S5: Average experimental mass (n=3 unless indicated otherwise) and the calculated average mass deviation for the glycoforms putatively identified from the bioreactor study analysed through ProA-MS. "VHS" indicates the presence of a "VHS" amino acid sequence tag as a N-terminal modification.

Putative Identification	Theoretical Mass	Average Experimental Mass	Average Mass Deviation
**Control Day 8**			
**Isotype 1**			
A2G0F/A2G1F	149987.9653	149985.4531	-16.7494
**Isotype 2**			
A2G1F/A2G1F	150150.1062	150147.4427	-17.7388
VHS+A2G0F/A2G0F	150149.1692	150147.4427	-11.4985
**Isotype 3**			
A2G1F/A2G2F	150312.2470	150310.2969	-12.9740
VHS+A2G0F/A2G1F	150311.3101	150310.2969	-6.7405
**Isotype 4**			
A2G2F/A2G2F	150474.3879	150472.9219	-9.7426
VHS+A2G1F/A2G1F	150473.4509	150472.9219	-3.5157
VHSx2+A2G0F/A2G0F	150472.5139	150472.9219	2.7112
**Isotype 5**			
VHS+A2G1F/A2G2F	150635.5917	150634.1771	-9.3913
VHSx2+A2G0F/A2G1F	150634.6548	150634.1771	-3.1711
**Control Day 10**			
**Isotype 1**			
A2G0F/A2G1F	149987.9653	149987.4531	-3.4150
**Isotype 2**			
A2G1F/A2G1F	150150.1062	150146.4792	-24.1560
VHS+A2G0F/A2G0F	150149.1692	150146.4792	-17.9158
**Isotype 3**			
A2G1F/A2G2F	150312.2470	150309.3438	-19.3150
VHS+A2G0F/A2G1F	150311.3101	150309.3438	-13.0815
**Isotype 4**			
A2G2F/A2G2F	150474.3879	150471.7135	-17.7727
VHS+A2G1F/A2G1F	150473.4509	150471.7135	-11.5459
VHSx2+A2G0F/A2G0F	150472.5139	150471.7135	-5.31909
**Isotype 5**			
VHS+A2G1F/A2G2F	150635.5917	150634.2031	-9.2184*
VHSx2+A2G0F/A2G1F	150634.6548	150634.2031	-2.9983*
**Low DO Day 8**			

**Isotype 1**			
A2G0F/A2G1F	149987.9653	149989.3958	9.5374
**Isotype 2**			
A2G1F/A2G1F	150150.1062	150149.0625	-6.9510
VHS+A2G0F/A2G0F	150149.1692	150149.0625	-0.71066
**Isotype 3**			
A2G1F/A2G2F	150312.2470	150310.7292	-10.0981
VHS+A2G0F/A2G1F	150311.3101	150310.7292	-3.8646
**Isotype 4**			
A2G2F/A2G2F	150474.3879	150472.9063	-9.8464
VHS+A2G1F/A2G1F	150473.4509	150472.9063	-3.6196
VHSx2+A2G0F/A2G0F	150472.5139	150472.9063	2.6073
**Isotype 5**			
VHS+A2G1F/A2G2F	150635.5917	150634.4948	-7.2822
VHSx2+A2G0F/A2G1F	150634.6548	150634.4948	-1.0620[*]
**Low DO Day 10**			
**Isotype 1**			
A2G0F/A2G1F	149987.9653	149987.3542	-4.0748
**Isotype 2**			
A2G1F/A2G1F	150150.1062	150148.9271	-7.8528
VHS+A2G0F/A2G0F	150149.1692	150148.9271	-1.6125
**Isotype 3**			
A2G1F/A2G2F	150312.2470	150311.5781	-4.4501
VHS+A2G0F/A2G1F	150311.3101	150311.5781	1.7835
**Isotype 4**			
A2G2F/A2G2F	150474.3879	150470.7813	-23.9684
VHS+A2G1F/A2G1F	150473.4509	150470.7813	-17.7417
VHSx2+A2G0F/A2G0F	150472.5139	150470.7813	-11.5148
**Isotype 5**			
VHS+A2G1F/A2G2F	150635.5917	150636.03125	2.9176[ǂ]
VHSx2+A2G0F/A2G1F	150634.6548	150636.03125	9.1379[ǂ]
**Low Temperature Day 8**			
**Isotype 1**			
A2G0F/A2G1F	149987.9653	149989.3125	8.9818
**Isotype 2**			
A2G1F/A2G1F	150150.1062	150149.6458	-3.0660
VHS+A2G0F/A2G0F	150149.1692	150149.6458	3.1744
**Isotype 3**			

A2G1F/A2G2F	150312.2470	150310.2969	-12.9740
VHS+A2G0F/A2G1F	150311.3101	150310.2969	-6.7405
**Isotype 4**			
A2G2F/A2G2F	150474.3879	150472.7917	-10.6079
VHS+A2G1F/A2G1F	150473.4509	150472.7917	-4.3811
VHSx2+A2G0F/A2G0F	150472.5139	150472.7917	1.8458
**Isotype 5**			
VHS+A2G1F/A2G2F	150635.5917	150634.1979	-9.2530
VHSx2+A2G0F/A2G1F	150634.6548	150634.1979	-3.0328
**Low Temperature Day 10**			
**Isotype 1**			
A2G0F/A2G1F	149987.9653	149987.19	-5.1860
**Isotype 2**			
A2G1F/A2G1F	150150.1062	150149.5417	-3.7597
VHS+A2G0F/A2G0F	150149.1692	150149.5417	2.4806
**Isotype 3**			
A2G1F/A2G2F	150312.2470	150311.2708	-6.4945
VHS+A2G0F/A2G1F	150311.3101	150311.2708	-0.2609
**Isotype 4**			
A2G2F/A2G2F	150474.3879	150473.1771	-8.0465
VHS+A2G1F/A2G1F	150473.4509	150473.1771	-1.8197
VHSx2+A2G0F/A2G0F	150472.5139	150473.1771	4.4072
**Isotype 5**			
VHS+A2G1F/A2G2F	150635.5917	150633.8516	-11.5523*
VHSx2+A2G0F/A2G1F	150634.6548	150633.8516	-5.3321*
**Low DO and Temperature Day 8**			
**Isotype 1**			
A2G0F/A2G1F	149987.9653	149988.2292	1.7590
**Isotype 2**			
A2G1F/A2G1F	150150.1062	150148.5729	-10.2116
VHS+A2G0F/A2G0F	150149.1692	150148.5729	-3.9713
**Isotype 3**			
A2G1F/A2G2F	150312.2470	150310.7813	-9.7516
VHS+A2G0F/A2G1F	150311.3101	150310.7813	-3.5180
**Isotype 4**			
A2G2F/A2G2F	150474.3879	150473.3490	-6.9043
VHS+A2G1F/A2G1F	150473.4509	150473.3490	-0.6775
VHSx2+A2G0F/A2G0F	150472.5139	150473.3490	5.5496

**Isotype 5**			
VHS+A2G1F/A2G2F	150635.5917	150634.0313	-10.3594
VHSx2+A2G0F/A2G1F	150634.6548	150634.0313	-4.1393
**Low Do and Temperature Day 10**			
**Isotype 1**			
A2G0F/A2G1F	149987.9653	149990.2031	14.9198
**Isotype 2**			
A2G1F/A2G1F	150150.1062	150150.0208	-0.5685
VHS+A2G0F/A2G0F	150149.1692	150150.0208	5.6719
**Isotype 3**			
A2G1F/A2G2F	150312.2470	150310.7969	-9.6476
VHS+A2G0F/A2G1F	150311.3101	150310.7969	-3.4141
**Isotype 4**			
A2G2F/A2G2F	150474.3879	150474.7604	2.4757
VHS+A2G1F/A2G1F	150473.4509	150474.7604	8.7027
VHSx2+A2G0F/A2G0F	150472.5139	150474.7604	14.930
**Isotype 5**			
VHS+A2G1F/A2G2F	150635.5917	150636.3177	4.8193
VHSx2+A2G0F/A2G1F	150634.6548	150636.3177	11.0396

[*] n=2

[ǂ] n=1
